# Changes in dental plaque following hospitalisation in a critical care unit: an observational study

**DOI:** 10.1186/cc12878

**Published:** 2013-09-04

**Authors:** Mishal Sachdev, Derren Ready, David Brealey, Jung Hyun Ryu, Georgia Bercades, Janette Nagle, Susana Borja-Boluda, Elisa Agudo, Aviva Petrie, Jean Suvan, Nikos Donos, Mervyn Singer, Ian Needleman

**Affiliations:** 1 Unit of Periodontology and International Centre for Evidence-Based Oral Health, UCL Eastman Dental Institute, 256 Gray's Inn Road, London WC1X 8LD, UK; 2 HPA, London Public Health Laboratory, Barts Health NHS Trust, Department of Infection, 3rd Floor Pathology & Pharmacy Building, 80 Newark Street, London E1 2ES, UK; 3 UCLH Bloomsbury Institute of Intensive Care Medicine, University College Hospital, 235 Euston Road, London, NW1 2BU, UK; 4 Biostatistics Unit, UCL Eastman Dental Institute, 256 Gray's Inn Road, London WC1X 8LD, UK

## Abstract

**Introduction:**

Previous research has suggested that deterioration in oral health can occur following hospitalisation. The impact of such deterioration could increase the risk of oral disease, reduce quality of life and increase the potential for healthcare-associated infections (HCAI) such as healthcare-associated pneumonia (HAP). However, the strength of the evidence is limited by, amongst other factors, the few observational studies published that assess oral health longitudinally. In view of the microbiological component of oral diseases and HCAIs, the objective of this study was to investigate the microbiological changes in dental plaque following hospitalisation in a Critical Care Unit (CCU): (1) total number of cultivable bacteria and (2) presence and changes in specific HAP pathogens.

**Methods:**

We conducted a prospective, longitudinal observational study in the CCU of University College Hospital, London. Study participants were recruited within 24 hours of admission. Dental plaque samples were collected from up to six sites per patient. The primary outcome was microbiological change from baseline to seven days with additional analysis for participants still present at day 14.

**Results:**

50 patients were recruited with 36 available for review at one week, with early discharge accounting for much of the loss to follow-up. The median total viable count of the plaque microbiota at baseline was 4.40 × 10^5 ^cfu/ml and increased at week one to 3.44 × 10^6 ^cfu/ml. The total viable microbe counts increased by a median of 2.26 × 10^6 ^cfu/ml from baseline to week one (95% CI: 3.19 × 10^6^, 1.24 × 10^7^) and this was statistically significant (*P *< 0.01). Specific HAP bacteria were detected in 26% of participants sampled, although accounted for a relatively low proportion of the total viable bacteria.

**Conclusion:**

Total bacterial count of dental plaque increases during hospitalisation in CCU. This finding, together with the colonisation of dental plaque by HAP bacteria strengthens the evidence for a deterioration in oral health in CCU and a risk factor for negative health and quality of life outcomes.

## Introduction

Current evidence suggests that oral health deteriorates following admission to hospital, particularly in the critical care setting [1]. This is highly relevant because of the possible impact of deterioration on development of oral disease, effect on quality of life and well-being and risk of HCAI.

In terms of oral health, we have shown in a systematic review that hospitalisation is associated with increased dental plaque accumulation, which leads to increased risk of inflammatory conditions in the mouth, such as periodontal disease [1]. In addition to local disease effects, oral inflammation has been shown to increase systemic inflammatory burden [2]. Mucositis and dry mouth have also been frequently reported, and, therefore, it is not surprising that poor oral health has been shown to reduce quality of life, comfort and well-being [3,4]. However, the strength of the evidence in this systematic review was limited by the few prospective, longitudinal, observational studies, their risk of bias and the use of outcome measures without full validity assessment.

Increasingly, poor oral health is implicated as a factor in the development of HCAIs such as healthcare-acquired pneumonia (HAP) [5-7]. If the oral biofilm provides a reservoir of respiratory pathogens that subsequently lead to pneumonia, then interventions to secure and maintain oral health should reduce the risk of HAP. Mechanical and chemical oral hygiene measures have been shown to reduce the rate of respiratory pathogen colonisation in the oral microbiota [8,9], thus reducing the risk for HAP amongst hospital inpatients. Although HAP-associated bacteria may enter the lungs in several different ways, aspiration of bacteria from the mouth and oropharynx is perhaps the most important mechanism of lower-airway infection in pneumonia pathogenesis [10]. Indeed, studies have reported a high degree of similarity of the microbiota upon comparing oral and respiratory samples in HAP [5,11,12].

Taken together, the evidence suggests that admission to a critical care unit (CCU) is associated with deterioration of oral health and that the impact of this deterioration is potentially a serious public health concern. Therefore, in view of the limited evidence available, we designed this study to investigate the effect of stay in CCU on dental plaque following the first seven days of hospitalisation in a CCU. The first part of the study reporting clinical outcomes was published previously [13], and this report describes the microbiological findings.

## Materials and methods

### Study design and setting

Prospective observational study in the CCU of University College Hospital, London. The unit offers high dependency and intensive care to a mixed population of medical and surgical patients. The unit has 35 beds and is managed by consultant-led multidisciplinary teams. Ethical approval was obtained from Central London Research Ethics Committee 3 (09/H0716/66).

### Participant selection and eligibility criteria

All patients admitted to the CCU were screened as potential participants to be included in this study within 24 hours of admission. Patient inclusion criteria were assessment within 24 hours of admission, minimum of six teeth present (dentures could be present but were not counted as teeth), at least 18 years of age, and not having been an inpatient within the previous one month. Exclusion criteria included edentulous patients and patient conditions that prevented assessment, for example, following major maxillofacial surgery.

The patient characteristics recorded were age, gender and nutritional status (nil by mouth, enteral tube-feeding, parenteral nutrition and oral nutrition); if supplementary oxygen was required, the mode of delivery was noted; and either self-ventilating (via nasal cannulae or facemask) or requiring mechanical ventilation (noninvasive or via an oral endotracheal tube or tracheostomy); and dependency for oral care (dependent or independent). Dependent participants were defined as those who were fully dependent on nursing staff for the provision of their dental hygiene, whilst independent patients conducted their own dental hygiene. Oral care policy for dependent patients was brushing teeth twice per day, 2% chlorhexidine gel three times per day, and sponge brushes with water twice per day.

### Consent/assent

Participants were recruited into the study within 24 hours of admission. Assent to participate was sought from a personal consultee, or, if absent, a professional consultee. Under such circumstances, formal assent could be delayed for up to 48 hours, and, if it was later withheld, participation in the study was discontinued and the patient's data destroyed.

### Assessment and plaque sampling

Dental plaque was sampled at representative Ramfjord teeth (FDI World Dental Federation notation 16, 21, 24, 36, 41 and 44) on their buccal surfaces using a sterile graduated periodontal probe (CPITN-C; DENTSPLY International, Weybridge, UK) and a halogen head torch. If teeth were missing or obscured by endotracheal tubes or were denture teeth, the adjacent tooth was sampled in a clockwise manner. As this was an observational study, there was no change to ongoing nursing interventions for oral healthcare implemented during this study. Sampling was organised to avoid affecting medical care and with the agreement of the nursing staff. Each assessment lasted a maximum of five minutes.

### Intervals of outcome assessment

Following baseline, the next examination was conducted at day 7 (± 2 days). The variable follow-up period was employed to allow some flexibility around the hospital and staff routines. A further assessment of plaque was conducted at day 14 (± 2 days) for those patients still present in CCU.

### Microbiology

The samples of dental plaque were placed in a single plastic cryopreservation transport tube (SARSTEDT Ltd, Beaumont Leys, UK) container with 1.0 ml of reduced transport fluid supplemented with 10% glycerol (cryoprotectant). The samples were stored immediately in a -70°C freezer until they were processed. The plaque samples were vortex-mixed for 60 seconds, and serial dilutions were prepared in sterile phosphate-buffered saline (Oxoid Ltd, Basingstoke, UK). Each dilution was inoculated (in duplicate) onto Fastidious Anaerobe Agar (E&O Laboratories, Bonneybridge, UK) supplemented with 5% defibrinated horse blood to determine the total number of cultivable bacteria in the specimen. Colonies were enumerated after five to seven days of incubation in an anaerobic cabinet at 37°C. The isolation and enumeration of organisms associated with HAP were achieved by inoculation of the dilutions onto the following selective media and incubation: (1) mannitol salt agar (aerobic incubation) for *Staphylococcus aureus*; (2) cetrimide agar (aerobic incubation) for *Pseudomonas aeruginosa*; (3) blood agar (incubated in 5% CO_2_/air) for *Streptococcus pneumoniae*; (4) bacitracin chocolate agar (incubated in 5% CO_2_/air) for *Haemophilus influenzae*; and (5) MacConkey agar (aerobic incubation) for *Klebsiella pneumoniae*, *Serratia marcescens*, *Proteus mirabilis*, *Escherichia coli *and *Enterobacter cloacae*.

After incubation, the various colony types on each medium were counted. Identification was completed by determining atmospheric growth requirement, Gram stain reaction, haemolysis, catalase, oxidase and coagulase reactions and optochin sensitivity. If these tests still proved equivocal, further species identification was carried out using the API strip identification system (API 20 and API 20 NE; bioMérieux UK Ltd, Basingstoke, UK).

### Sample size calculation

Study power was calculated for clinical dental plaque changes [13]. Using a paired *t*-test at 90% power and a significance level of 0.05, 35 participants would be required to detect as statistically significant at least one-half unit change in full-mouth plaque scores over seven days, assuming a SD of the differences of 0.88. A sample size of 50 participants was agreed to allow expected losses to follow-up within the CCU.

### Data analysis

All statistical analysis was carried out using SPSS software version 20 (SPSS, Inc, Chicago, IL, USA). Initially, summary measures were described in terms of frequency, median and ranges for baseline in weeks 1 and 2. Thereafter the overall proportions of cultivable plaque bacteria which contained specific HAP-associated bacteria were described. Nonparametric methods were used to assess changes in the microbiology from baseline to weeks 1 and 2, in the form of a Wilcoxon signed-rank test. Wherever possible, data were analysed to investigate the impact of potential prognostic variables, such gender and dependency for oral care, by use of a Mann-Whitney *U *test.

## Results

### Characteristics of study participants

The baseline characteristics of participants are given in Table 1. Most of these patients were nil by mouth for their nutrition (65%) and were self-ventilating with nasal cannulae (62%). There were also more patients who were dependent on the nursing teams for their oral care (56%) than those who were able to complete their own oral care (44%). Plaque samples were collected from 50 patients at baseline. At week 1, 36 of these patients were still present for microbial sampling, and 10 patients remained for sampling at week 2. The causes of losses to follow-up were early discharge or death (baseline to week 1, *n *= 14; week 1 to week 2, *n *= 16).

**Table 1:** Baseline characteristics

Characteristics	Number of patients	Percentage
Gender		
Female	27	54%
Male	23	46%
		
Nutritional status		
Nil by mouth	34	65%
Oral	12	24%
Enteral	3	6%
Parenteral	1	2%
		
Dependency of oral care		
Dependent	28	56%
Independent	22	44%
		
Type of ventilation		
Nasal	31	62%
Endotracheal	12	24%
Self-ventilated	4	8%
Facemask	3	6%
		
Medications		
Antibiotics	38	76%
Antacids	33	66%
Antifungals	3	6%

### Total viable microbial counts

The median total viable count of the plaque microbiota at baseline was 4.40 × 10^5 ^colony-forming units (cfu)/ml (range when detected of 8.00 × 10^3 ^cfu/ml to 4.56 × 10^7 ^cfu/ml) (Table 2). The median total viable count of the plaque microbiota at week 1 was 3.44 × 10^6 ^cfu/ml (range when detected of 1.40 × 10^4 ^cfu/ml to 4.46 × 10^7 ^cfu/ml), and for week 2 it was 2.99 × 10^5 ^cfu/ml (range when detected of 1.00 × 10^5 ^cfu/ml to 4.50 × 10^7 ^cfu/ml). The median was taken as a descriptive summary measure because the data were skewed. The detectable limit of bacteria was 4 × 10^2 ^cfu/ml.

**Table 2:** Median change in total viable microbe{AU: "microbe" correct here? As meant?} counts between baseline and follow-up visits per sample

Baseline	Increase between baseline and week 1	Increase between weeks 1 and 2
(*n *= 50)	(*n *= 36)	(*n *= 10)
4.40 × 10^5 ^cfu/ml	2.26 × 10^6 ^cfu/ml (*P *< 0.01)	-7 × 10^3 ^cfu/ml (*P *< 0.77)

### Change in total viable counts between visits

The total viable microbe counts increased by a median of 2.26 × 10^6 ^cfu/ml from baseline to week 1 (95% CI: 3.19 × 10^6^, 1.24 × 10^7^), and this change was statistically significant by the Wilcoxon signed-rank test (*P *= 0.01). The change from week 1 to week 2 was not statistically significant (*P *= 0.77), although it was based on only ten patients.

### Hospital-acquired pneumonia (HAP)-associated bacteria

**Figure 1:**
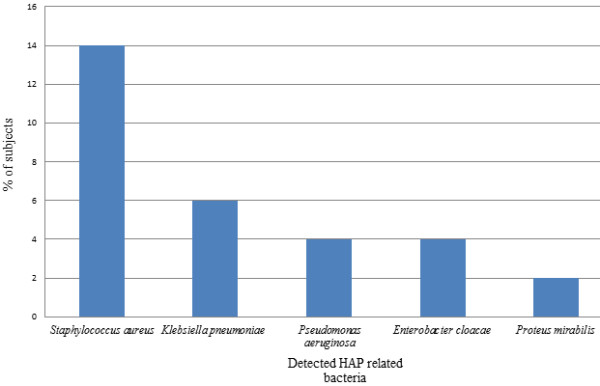
**Proportion of patients colonised by specific bacteria associated with healthcare-associated pneumonia (HAP)**.

Overall, the dental plaques of 13 (26%) of 50 patients were colonised by HAP-associated pathogens. The HAP-specific bacteria examined included *Staphylococcus aureus*, *Klebsiella pneumoniae*, *Pseudomonas aeruginosa*, *Enterobacter cloacae*, *Proteus mirabilis*, *Serratia *sp., *Escherichia coli*, *Haemophilus influenzae *and *Streptococcus pneumoniae *(Figure 1). There was little qualitative difference between baseline and week 1, so the presence of bacteria was reported at either baseline or week 1.

The proportion of the total cultivable plaque bacteria which comprised *Staphylococcus aureus *ranged from 0.02% to a maximum of 8.14% when detected. Seven (14%) of fifty patients had a detectable level of their total viable microbiota comprising *Staphylococcus aureus*. The proportion of the total cultivable plaque bacteria which comprised *K. pneumoniae*, ranged from less than 0.001% to a maximum of 0.02%, with three (6%) of fifty patients having a detectable level of their total viable microbiota comprising *K. pneumoniae*. Two (4%) of 50 patients harboured *Enterobacter cloacae*, which comprised 0.06% and 0.41% of their total viable oral microbiota, respectively. One of the 50 patients harboured *Proteus mirabilis*. Interestingly, *Serratia *sp., *Escherichia coli, H. influenzae *and *Streptococcus pneumoniae *were not detected. No comparisons were conducted between sample dates, as the level of HAP pathogen carriage was low. The level of the total cultivable plaque microbes which comprised yeasts ranged from 0.11% to a maximum of 2.28% when detected, with 17 (34%) of 50 patients having a detectable level.

### Relationship of change in total viable count to clinical characteristics from baseline to week 1

**Figure 2:**
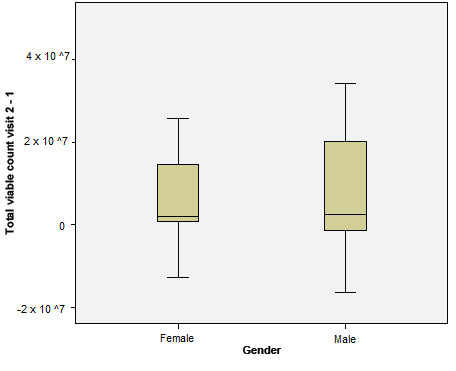
**Change in median total viable count in relation to gender from baseline to week 1**.

**Figure 3:**
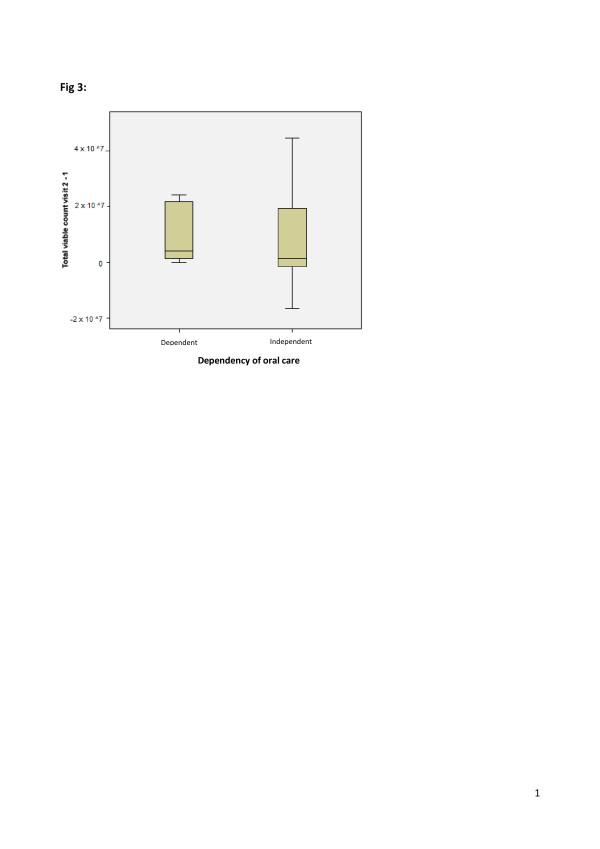
**Change in median total viable count in relation to dependency of oral care from baseline to week 1**.

Subgroup sizes were judged to be too small to test many of the demographic and clinical characteristics. When evaluating total bacterial count in conjunction with gender and dependency for oral care, however, we found that there was no evidence of an effect of either variable by Mann-Whitney *U *test on the change in the total viable bacterial count between baseline and week 1 (gender, *P *= 0.20 (Figure 2); dependency for oral care, *P *= 0.33 (Figure 3)).

## Discussion

### Key findings

The key findings of this observational study are that the median total viable dental plaque increased from CCU admission to week 1 and that the dental plaque of 26% of patients was colonised by HAP-associated organisms.

### Strengths and weaknesses of the study

Key strengths of the study include the prospective nature of the study, the speed of participant recruitment within 24 hours and careful microbial sampling and culture. The main limitation of this study is that there was a lack of a true control group. However, it is difficult to design a meaningful comparison. Investigating patients following admission to another hospital ward or nursing home, for example, might provide a valuable evaluation of a different setting but would not be a true control.

Another limitation is sample size. We calculated study power for the primary clinical outcome (dental plaque index), and recruitment met this target [13]. However, the number of patients may have been too small to detect changes in microbiological outcomes such as the colonisation of dental plaque by HAP pathogens. It is also possible that sampling of dental plaque for assay might have affected the biofilm, both quantitatively and qualitatively. We considered this issue prior to the study, and, as plaque was required for the culture, there was no straightforward method to overcome it. However, we sought to reduce the effect by disrupting the biofilm as little as possible with the use of a 0.5-mm-diameter tip periodontal probe. This sampling method was thought to be less invasive than other sampling techniques that employ dental scalers or paper points. However, collection of a larger amount of dental plaque might have resulted in different findings. In order to collect more dental plaque, follow-up assessments would need to be conducted on different teeth, thereby introducing the potential for greater variability.

As we did not record the incidence of HAP, we do not know whether those participants who harboured HAP-associated bacteria developed HAP infection, although colonisation of the oral biofilm by respiratory pathogens may be a surrogate measure for risk of infection [11,14,15]. A few published studies have considered oral and lung colonisation in a CCU setting and then related this to the prevalence of HAP [5,11,12]. In those studies, a wide range of association (0% to 88%) was found when bacteria isolated from the oropharyngeal region were examined for species similar to those found by fibre optic bronchoscopy or bronchoalveolar lavage of the lungs.

### Assessment of findings in relation to other studies

The findings of our study in general are supported by other reports that show a significant increase in the total viable dental plaque from within 24 hours of hospital admission (at baseline) to a follow-up assessment at one week [11,12,16]. In this study population, specific HAP bacteria were detected to a similar extent (26%) when compared to other studies (30% to 60%). Nonetheless, the specific bacteria accounted for a relatively low proportion of the total viable bacteria, with a maximum proportion of 8.14% for *Staphylococcus aureus*, which is in keeping with findings of other similar studies [16,17]. The differences may be due to methodology such as cryopreservation of samples prior to culture, unlike other previous studies [16,18-20]. In this study, we investigated the presence and absence of HAP-related bacteria together with the proportion of the total viable bacterial count, and this has been considered by only two other culture-based studies [16,21]. Our analysis was completed prospectively with a clear plan regarding which bacteria to assess. Other studies were either conducted retrospectively, or were unclear regarding which bacteria were selected for analysis from the outset [11,18,22,23]. In studies in which only positive findings were reported, the representativeness of these data are unclear.

### Implications of the study for clinicians and policymakers

This study adds to the growing body of evidence that oral health deteriorates following hospitalisation [1,13,21,24]. Because deteriorating oral health is associated with a greater risk of healthcare-associated infections [7] and reduced quality of life [3,4], there are important implications for overall healthcare.

Factors that may account for this deterioration include independent patients' becoming dependent upon hospital staff for oral care, which is further aggravated by the absence of training or equipment to deliver effective oral hygiene; the low priority of oral care in CCUs; and the lack of clear guidelines on oral care delivery [1,25,26]. The hospital routine for oral care included sponge toothettes and topical chlorhexidine in dependent patients, and independent patients were encouraged to continue their own oral hygiene routine. Our results showed the amount of dental plaque significantly increased within CCUs. It is possible that this deterioration in oral health would be even greater in hospital units with less intensive nursing care and support.

Even though guidelines are available in this field [27-29], oral care may be ineffective and poorly followed and may not be evidence-based [25]. Therefore, improvements in healthcare need to be based on both better evidence for effective interventions and for their successful implementation in this challenging environment.

### Future research

Priorities for further research should include prospective studies of the relationship between colonisation of the dental plaque biofilm by HAP-associated bacteria and the development of HAP itself. It would be helpful to broaden the setting of these studies to non-CCU and long-term care units.

Additional carefully conducted intervention trials are still required to identify the most promising interventions or bundles of care. As mentioned previously, however, implementation studies will be an essential stage of future research to translate promising interventions into effective care.

## Conclusions

Hospitalisation is associated with a substantial increase in the total viable count of dental plaque bacteria. In our present study, we found that 26% of patients were colonised by hospital-associated pneumonia pathogens.

This study adds to the evidence showing the deterioration in oral health following hospitalisation in CCUs. This finding is extremely important for both individual and public health. Existing evidence clearly indicates that poor oral health reduces quality of life and well-being, in addition to contributing to healthcare-associated infections [7,30,31]. Priorities for further research should therefore include evaluating oral health changes in hospital settings outside the CCU, identifying the best oral care interventions in different settings and developing effective implementation strategies for care pathways directed towards maintaining oral health.

## Key messages

• Total viable bacterial count of dental plaque increases following admission to the CCU.

• We found that 26% of patients were colonised by hospital-associated pneumonia pathogens.

• This study adds to the growing evidence of deterioration in oral health in the CCU.

• Deteriorating oral health increases the risks for oral disease, negative effects on quality of life and healthcare-associated infections.

• Existing standards of oral care in the CCU do not maintain oral health.

## Abbreviations

CCU: Critical Care Unit; HCAI: Healthcare-associated infection; HAP: Healthcare-associated pneumonia.

## Competing interests

The authors declare that they have no competing interests.

## Authors' contributions

IN conceived the study and was chiefly responsible for the design and writing of the manuscript. DR served as the microbiology expert and conducted some assays. MS carried out microbiological assays, supported the conduct of the study and wrote the first draft of the manuscript. DB provided CCU coordination, expert knowledge and input into the design and reporting of study. JHR acted as CCU study coordinator and sampler of dental plaque. GB, SB and JN were study examiners. EA contributed to the first design of the protocol and examiner training. AP was the biostatistician and supported statistical design and analysis. JS participated in study design and research governance. ND provided expert periodontal input into the design of study. MS provided expert intensive care medicine input. All authors read and approved the final manuscript.

## References

[B1] Terezakis E Needleman I Kumar N Moles D Agudo E The impact of hospitalization on oral health: a systematic review J Clin Periodontol 2011 17 628 636 21470276 10.1111/j.1600-051X.2011.01727.x

[B2] Tonetti MS D'Aiuto F Nibali L Donald A Storry C Parkar M Suvan J Hingorani AD Vallance P Deanfield J Treatment of periodontitis and endothelial function N Engl J Med 2007 17 911 920 17329698 10.1056/NEJMoa063186

[B3] Llewellyn CD Warnakulasuriya S The impact of stomatological disease on oral health-related quality of life Eur J Oral Sci 2003 17 297 304 12887394 10.1034/j.1600-0722.2003.00057.x

[B4] Yu DS Lee DT Hong AW Lau TY Leung EM Impact of oral health status on oral health-related quality of life in Chinese hospitalised geriatric patients Qual Life Res 2008 17 397 405 18264796 10.1007/s11136-008-9314-9

[B5] Heo SM Haase EM Lesse AJ Gill SR Scannapieco FA Genetic relationships between respiratory pathogens isolated from dental plaque and bronchoalveolar lavage fluid from patients in the intensive care unit undergoing mechanical ventilation Clin Infect Dis 2008 17 1562 1570 18991508 10.1086/593193 PMC3582026

[B6] Scannapieco FA Pneumonia in nonambulatory patients: the role of oral bacteria and oral hygiene J Am Dent Assoc 2006 17 Suppl 21S 25S A published erratum appears in *J Am Dent Assoc *2008, **139**:252. 17012732 10.14219/jada.archive.2006.0400

[B7] Azarpazhooh A Leake JL Systematic review of the association between respiratory diseases and oral health J Periodontol 2006 17 1465 1482 16945022 10.1902/jop.2006.060010

[B8] Chan EY Ruest A Meade MO Cook DJ Oral decontamination for prevention of pneumonia in mechanically ventilated adults: systematic review and meta-analysis BMJ 2007 17 889 17387118 10.1136/bmj.39136.528160.BE PMC1857782

[B9] Needleman IG Hirsch NP Leemans M Moles DR Wilson M Ready DR Ismail S Ciric L Shaw MJ Smith M Garner A Wilson S Randomized controlled trial of toothbrushing to reduce ventilator-associated pneumonia pathogens and dental plaque in a critical care unit J Clin Periodontol 2011 17 246 252 21223352 10.1111/j.1600-051X.2010.01688.x

[B10] Scannapieco FA Role of oral bacteria in respiratory infection J Periodontol 1999 17 793 802 10440642 10.1902/jop.1999.70.7.793

[B11] Garrouste-Ortegas M Chevret S Arlet G Marie O Rouveau M Popoff N Schlemmer B Oropharyngeal or gastric colonization and nosocomial pneumonia in adult intensive care unit patients: a prospective study based on genomic DNA analysis Am J Respir Crit Care Med 1997 17 1647 1655 10.1164/ajrccm.156.5.96-04076 9372689

[B12] Fourrier F Duvivier B Boutigny H Rourrel-Delvallez M Chopin C Colonization of dental plaque: a source of nosocomial infections in intensive care unit patients Crit Care Med 1998 17 301 308 9468169 10.1097/00003246-199802000-00032

[B13] Needleman I Hyun-Ryu J Brealey D Sachdev M Moskal-Fitzpatrick D Bercades G Nagle J Lewis K Agudo E Petrie A Suvan J Donos N Singer M The impact of hospitalization on dental plaque accumulation: an observational study J Clin Periodontol 2012 17 1011 1016 22957747 10.1111/j.1600-051X.2012.01939.x

[B14] Rello J Ollendorf DA Oster G Vera-Llonch M Bellm L Redman R Kollef MH VAP Outcomes Scientific Advisory Group Epidemiology and outcomes of ventilator-associated pneumonia in a large US database Chest 2002 17 2115 2121 12475855 10.1378/chest.122.6.2115

[B15] Nilsson A Björkman P Welinder-Olsson C Widell A Persson K Clinical severity of *Mycoplasma pneumoniae *(MP) infection is associated with bacterial load in oropharyngeal secretions but not with MP genotype BMC Infect Dis 2010 17 39 20184731 10.1186/1471-2334-10-39 PMC2837002

[B16] Scannapieco FA Stewart EM Mylotte JM Colonization of dental plaque by respiratory pathogens in medical intensive care patients Crit Care Med 1992 17 740 745 1597025 10.1097/00003246-199206000-00007

[B17] Fourrier F Dubois D Pronnier P Herbecq P Leroy O Desmettre T Pottier-Cau E Boutigny H Di Pompéo C Durocher A Roussel-Delvallez M PIRAD Study Group Effect of gingival and dental plaque antiseptic decontamination on nosocomial infections acquired in the intensive care unit: a double-blind placebo-controlled multicenter study Crit Care Med 2005 17 1728 1735 16096449 10.1097/01.ccm.0000171537.03493.b0

[B18] Terpenning M Bretz W Lopatin D Langmore S Dominguez B Loesche W Bacterial colonization of saliva and plaque in the elderly Clin Infect Dis 1993 17 Suppl 4 S314 S316 8324138 10.1093/clinids/16.supplement_4.s314

[B19] Sumi Y Miura H Michiwaki Y Nagaosa S Nagaya M Colonization of dental plaque by respiratory pathogens in dependent elderly Arch Gerontol Geriatr 2007 17 119 124 16723159 10.1016/j.archger.2006.04.004

[B20] Ewan V Perry JD Mawson T McCracken G Brown AN Newton J Walls A Detecting potential respiratory pathogens in the mouths of older people in hospital Age Ageing 2010 17 122 125 19749149 10.1093/ageing/afp166 PMC2794360

[B21] Fourrier F Duvivier B Boutigny H Roussel-Delvallez M Chopin C Colonization of dental plaque: a source of nosocomial infections in intensive care unit patients Crit Care Med 1998 17 301 308 9468169 10.1097/00003246-199802000-00032

[B22] Russell SL Boylan RJ Kaslick RS Scannapieco FA Katz RV Respiratory pathogen colonization of the dental plaque of institutionalized elders Spec Care Dentist 1999 17 128 134 10860077 10.1111/j.1754-4505.1999.tb01413.x

[B23] Terpenning M Bretz W Lopatin D Langmore S Dominguez B Loesche W Bacterial colonization of saliva and plaque in the elderly Clin Infect Dis 1993 17 Suppl 4 S314 S316 8324138 10.1093/clinids/16.supplement_4.s314

[B24] Munro CL Grap MJ Elswick RK Jr McKinney J Sessler CN Hummel RS Oral health status and development of ventilator-associated pneumonia: a descriptive study Am J Crit Care 2006 17 453 460 16926366

[B25] Rello J Koulenti D Blot S Sierra R Diaz E De Waele JJ Macor A Agbaht K Rodriguez A Oral care practices in intensive care units: a survey of 59 European ICUs Intensive Care Med 2007 17 1066 1070 17384927 10.1007/s00134-007-0605-3

[B26] Berry AM Davidson PM Masters J Rolls K Systematic literature review of oral hygiene practices for intensive care patients receiving mechanical ventilation Am J Crit Care 2007 17 552 563 17962500

[B27] Fiske J Griffiths J Jamieson R Manger D British Society for Disability and Oral Health Working Group Guidelines for oral health care for long-stay patients and residents Gerodontology 2000 17 55 64 11203515 10.1111/j.1741-2358.2000.00055.x

[B28] Tablan OC Anderson LJ Besser R Bridges C Hajjeh R CDC; Healthcare Infection Control Practices Advisory Committee Guidelines for preventing health-care-associated pneumonia, 2003: recommendations of CDC and the Healthcare Infection Control Practices Advisory Committee MMWR Recomm Rep 2004 17 RR-3 1 36 15048056

[B29] Department of Health, NHS Trust Saving Lives: Reducing Infection, Delivering Clean and Safe Care (High Impact Intervention No 5): Care Bundle for Ventilated Patients (or Tracheostomy Where Appropriate). London: Author 2007

[B30] Locker D Matear D Stephens M Jokovic A Oral health-related quality of life of a population of medically compromised elderly people Community Dent Health 2002 17 90 97 12146588

[B31] Duke RL Campbell BH Indresano AT Eaton DJ Marbella AM Myers KB Layde PM Dental status and quality of life in long-term head and neck cancer survivors Laryngoscope 2005 17 678 683 15805880 10.1097/01.mlg.0000161354.28073.bc

